# Extracellular ATP and Imbalance of CD4+ T Cell Compartment in Pediatric COVID-19

**DOI:** 10.3389/fcimb.2022.893044

**Published:** 2022-05-18

**Authors:** Constanza Russo, Silvina Raiden, Silvia Algieri, Norberto De Carli, Carolina Davenport, Mariam Sarli, María José Bruera, Vanesa Seery, Inés Sananez, Nancy Simaz, Carola Bayle, Valeria Nivela, Fernando Ferrero, Jorge Geffner, Lourdes Arruvito

**Affiliations:** ^1^ Instituto de Investigaciones Biomédicas en Retrovirus y SIDA, Facultad de Medicina, Universidad de Buenos Aires- Consejo Nacional de Investigaciones Científicas y Técnicas, Ciudad Autónoma de Buenos Aires, Argentina; ^2^ Departamento de Medicina, Hospital General de Niños Pedro de Elizalde, Ciudad Autónoma de Buenos Aires, Argentina; ^3^ Servicio de Pediatría, Hospital Nacional Profesor Alejandro Posadas, Buenos Aires, Argentina; ^4^ Servicio de Pediatría Clínica del Niño de Quilmes, Buenos Aires, Argentina; ^5^ Unidad de Terapia Intensiva Pediátrica, Hospital Nacional Profesor Alejandro Posadas, Buenos Aires, Argentina; ^6^ Departamento de Emergencias Pediátrica, Hospital Nacional Profesor Alejandro Posadas, Buenos Aires, Argentina

**Keywords:** COVID-19, children, extracellular ATP, Tregs (regulatory T cells), TH17 cells

## Abstract

Severe COVID-19 in children is rare, but the reasons underlying are unclear. Profound alterations in T cell responses have been well characterized in the course of adult severe COVID-19, but little is known about the T cell function in children with COVID-19. Here, we made three major observations in a cohort of symptomatic children with acute COVID-19: 1) a reduced frequency of circulating FoxP3+ regulatory T cells, 2) the prevalence of a TH17 polarizing microenvironment characterized by high plasma levels of IL-6, IL-23, and IL17A, and an increased frequency of CD4+ T cells expressing ROR-γt, the master regulator of TH17 development, and 3) high plasma levels of ATP together with an increased expression of the P2X7 receptor. Moreover, that plasma levels of ATP displayed an inverse correlation with the frequency of regulatory T cells but a positive correlation with the frequency of CD4+ T cells positive for the expression of ROR-γt. Collectively, our data indicate an imbalance in CD4+ T cell profiles during pediatric COVID-19 that might favor the course of inflammatory processes. This finding also suggests a possible role for the extracellular ATP in the acquisition of an inflammatory signature by the T cell compartment offering a novel understanding of the involved mechanisms.

## Introduction

Epidemiological data from Argentina as of January 3, 2022 have reported more than 480,000 confirmed cases under 20 years and 0.05% death. It is well known that COVID-19 is less severe in children compared with adults (Lu et al., 2020; Ludvigsson, 2020; Boppana et al., 2021). Several hypotheses have been proposed to explain why children are protected from severe outcomes, including differences in the expression of angiotensin-converting enzyme 2 (ACE-2) (Bunyavanich et al., 2020), the receptor for viral entry, a more robust innate immune response in the early phase of the infection, and trained innate immunity related to the protection offered by vaccination and viral infections in childhood (de Candia et al., 2021; Pierce et al., 2021; Vono et al., 2021; Yonker et al., 2021). However, in some children SARS-CoV-2 triggers a dysregulated hyperinflammatory process and severe COVID-19 (Diorio et al., 2020; Sananez et al., 2021; Vella et al., 2021). Previous comorbidities increased the risk for severe disease, but it can also occur in healthy children (Funk et al., 2022).

Perhaps reflecting that children with COVID-19 usually develop a mild or asymptomatic disease, most studies directed to characterize the pathogenesis of SARS-CoV-2 infection have been performed in adults. Severe COVID-19 in adults is associated with an overactive inflammatory response with macrophages and neutrophils playing a critical role. Consistent with the ability of CD4+ T cells to regulate the recruitment and function of phagocytes in peripheral tissues (De Biasi et al., 2020; Grant et al., 2021), severe COVID-19 in adults have shown to be associated with changes in the function of two different CD4+ T cell profiles; FoxP3+ regulatory T cells (Tregs) and TH17 cells. While it is clear that severe COVID-19 is related to the induction of a TH17 response (De Biasi et al., 2020; Rupp et al., 2021; Zhao et al., 2021), conflicting results have been published regarding Tregs. Some studies have described an increased frequency of Tregs and the acquisition of an activated phenotype in severe COVID-19 patients (Neumann et al., 2020; Galvan-Pena et al., 2021; Rupp et al., 2021; Vick et al., 2021). By contrast, other studies reported a reduction in the frequency of Tregs in hospitalized patients (Qin et al., 2020; Wang et al., 2020; Sadeghi et al., 2021).

Recent studies have shown that extracellular ATP acts not only as a damage-associated molecular pattern required for inflammasome activation (Karmakar et al., 2016), but it is also able to modulate the function of CD4+ T cells through the activation of purinergic receptors promoting both the development of TH17 cells as well as the inhibition of Tregs (Atarashi et al., 2008; Zhao et al., 2016; D’Addio et al., 2018). Interestingly, it has been proposed that the blockade of purinergic receptors might represent a therapeutic option to prevent the progression of severe COVID-19 (Di Virgilio et al., 2020; Kanthi et al., 2020; Illes, 2021). Here, we studied the frequency of Tregs and TH17 in children with COVID-19. We also determined the profile of plasma cytokines and analyzed whether changes in plasma levels of ATP might be associated to the imbalance in the CD4+ T cell compartment observed in children with COVID-19.

## Subjects and Methods

### Study Population

This study was conducted in Buenos Aires City and Buenos Aires province, Argentina, between October 2020 and July 2021. We recruited girls and boys aged between 2 years and 12 years admitted to the Hospital General de Niños Pedro de Elizalde, Hospital Nacional Prof. Alejandro Posadas and Clínica del Niño de Quilmes. Patients with active SARS-CoV-2 infection confirmed by PCR of nasopharyngeal swabs (n=84) were included. Disease severity was classified according to the Health Ministry from Argentina and the World Health Organization interim guidance. Non-severe children (n=67) had mild or moderate disease. Severe children (n=17) had severe pneumonia, cough, difficulty in breathing, respiratory distress and/or lethargy and convulsions. Our control cohort included 42 children age and sex matched that were admitted to the hospitals for routine screening and/or scheduled surgery. They had no history of recent respiratory infection or close-contact and they were negative for IgM and IgG antibodies directed to SARS-CoV-2. Characteristics of cohorts are shown in **Table 1**.

**Table 1:** Characteristics of children with COVID-19 and healthy controls.

			COVID-19	
		Control	Non-severe	Severe
		n=42	n=67	n=17
**Age, y, median (range)**		7 (2-9)	3 (1-7)	4 (2-7)
**Female, n (%)**		17 (41)	26 (39)	11 (65)
**Days after symptom onset (range)**		N/A	2 (1-4)	3 (2-5)
**SARS-CoV-2 PCR positive**^**a**^**, n (%)**		N/A	67 (100)	17 (100)
**SARS-CoV-2 IgG antibody positive, n (%)**		0	31 (46)	9 (53)
**SARS-CoV-2 IgM antibody positive, n (%)**		0	37 (55)	11 (65)
**Comorbidities, n (%)**				
	None	39 (93)	32 (48)	8 (47)
	Heart disease	0	3 (4)	1 (6)
	Lung disease	0	4 (6)	2 (12)
	Prematurity	0	2 (3)	0
	Haematological disease	0	4 (6)	0
	Cancer	0	7 (10)	2 (12)
	Obesity	3 (7)	3 (4)	2 (12)
	Undernutrition	0	11 (16)	2 (12)
	Diabetes	0	3 (4)	3 (18)
	Genetics disorders	0	2 (3)	2 (12)
	Neurological	0	7 (10)	2 (12)
**Co-infections** ^**b**^ **, n (%)**		0	12 (18)	8 (47)
**Pneumonia, n (%)**		0	9 (13)	10 (59)
**PICU admission, n (%)**		0	4 (6)	13 (76)
**Mechanical ventilation, n (%)**		0	0	8 (47)

y, years; PICU, pediatric intensive care unit.

a at time of blood collection.

b Types of bacterial co-infection includes urinary tract infections (E. coli and K. pneumoniae), catheter-related infection with bloodstream infection (S. hominis), sepsis (P. Aeruginosa), and dysentery (Shigella). Viral co-infections include rhinovirus and respiratory syncytial virus infections.

### Blood Samples Processing

0.5-1 mL of whole blood samples were obtained within 1-3 days of hospital admission. After being centrifuged for 10 min at 1000 rpm, plasma was separated and stored at -80°C until used.

### Isolation of Peripheral Blood Mononuclear Cells

Peripheral blood mononuclear cells (PBMCs) were obtained from blood samples by Ficoll-Hypaque gradient centrifugation (GE Healthcare Life Sciences, Uppsala, Sweden). PBMCs were washed twice, and suspended in complete culture medium (RPMI 1640, Sigma-Aldrich, St. Louis, MO, USA) supplemented with 10% heat-inactivated fetal bovine serum (Natocor, Villa María, Córdoba, Argentina), 2 mM L-glutamine, 100U/mL penicillin and 0.1 mg/mL streptomycin (Sigma-Aldrich, St. Louis, MO, USA).

### CD4+ T Cell Isolation

CD4**+** T cells were enriched from whole blood by using the RosetteSep human CD4+ T cells enrichment cocktail (Stem Cell Technologies, Vancouver, BC, Canadá), following the manufacturer’s protocols. After washing, cells were suspended in complete culture medium. The purity determined by flow cytometry was always >97%.

### Flow Cytometry

Freshly isolated PBMCs (1x10^6^ cells) were stained with anti-CD4 PercP, anti-CD25 BV510 and anti-CD39 APC-Cy7; all from Biolegend (San Diego, CA, USA). Intracellular staining using anti-FoxP3 Alexa Fluor 488 (BD Biosciences, San Diego, CA, USA) and anti-ROR-γt PE (R&D, Minneapolis, MN, USA) was performed on fixed and permeabilized cells following the manufacturer´s instructions. Isotype-matched antibodies were used as control. Statistical analyses were based on at least 100,000 events gated on the population of interest. Data were acquired using a FACSCanto II (Becton Dickinson, San Diego, CA, USA) and analyzed with FlowJo.v10.6.2 (Ashland, OR, USA).

### LegendPlex Assay

Levels of cytokines in plasma (IL-1β, IL-6, IL-23, IL-17A, IL-2, IL-18 and IL-10) and in supernatant cultures from purified CD4+ T cells (IL-2, IFN-γ, TNF-α, IL-17A, IL-5, IL-13, IL-9 and IL-10) were analyzed by multiplex flow cytometric bead array according to the manufacturer’s instructions (Biolegend, San Diego, CA, USA).

### ATP Measurement

To measure the release of ATP from cells, 5x10^6^/mL PBMCs were stimulated with anti-CD2/CD3/CD28 coated beads (0.3 µg/mL, Miltenyi Biotec, Bergisch Gladbach, Germany) during 4 min and the supernatant culture were collected. The ATP levels in supernatant culture and/or the levels in plasma from donors were quantified using the CellTiter-Glo reagent (Promega, Madison, WI, USA) according to manufacturer’s instructions by luminometry (SpectraMax i3X, Molecular Devices, San José, CA, USA). To calculate the ATP concentration, a standard curve was plotted, and a regression analysis was applied.

### Confocal Microscopy

Briefly, purified CD4+ T cells were incubated with anti-CD4 Alexa Fluor 488 (Biolegend, San Diego, CA, USA). After washed, cells were fixed for 20 min at 4°C and then permeabilized with PBS-BSA 2%-Triton- X-100 0.3% for 20 min at room temperature. Then, cells were washed and stained with anti-P2X7 receptor (P2X7R) Alexa Fluor 647 (dil 1:50, Biolegend, San Diego, CA, USA) overnight at 4°C. Afterward, cells were washed twice and allowed to adhere on polylysine-coated coverslips. After washing, cells were fixed with 4% paraformaldehyde in PBS for 15 min at 4°C, and treated with 10 mM glycine for 10 min at room temperature. The coverslips mounted with DAPI Fluoroshield^®^ (Sigma-Aldrich, St. Louis, MO, USA) were examined under a confocal microscope (ZEISS LSM 800, Oberkochen, Germany).

### Quantitative RT-PCR

Total RNA was extracted from PBMCs using TRIzol^®^ reagent (Carlsbad, CA, USA) following manufacturer’s instructions. Subsequently, RNA was treated with RQ1 RNAse-free DNAse (Promega, Madison, WI, USA) and reverse transcripted using M-MLV Reverse Transcriptase (Promega, Madison, WI, USA). PCR analysis for P2X7R was performed with a real-time PCR detection system (StepONE-Plus Applied Biosystems, Waltham, MA USA) using 5× HOT FIREPol^®^ EvaGreen^®^ qPCR Mix Plus (ROX) (Solis BioDyne Corp, Tartu, Estonia) as a fluorescent DNA-binding dye. GAPDH was used as housekeeping gene. The primer sets used for amplification were as follows: P2X7R-F 5’-tccagtaactgctgtcgctc -3’ and P2X7R-R 5’- tggactcgcacttcttcctg-3’; GAPDH-F 5’-cgaccactttgtcaagctca-3’ and GAPDH-R 5’-ttactccttggaggccatgt-3’. Primer sets yielded a single product of the correct size. The relative mRNA expression level was calculated using 2^-ΔCt^, and the data were normalized according to GAPDH mRNA levels. The results are presented as a value relative to the control value.

### T Cells Culture

Purified CD4+ T cells **(**1x10^6^/mL) from donors were stimulated with anti-CD2/CD3/CD28 coated beads (0.035 µg/mL, Miltenyi Biotec, Bergisch Gladbach, Germany) and treated or not with 2´(3´)-O-(4-benzoylbenzoyl) ATP (100 µM, BzATP, P2X7R agonist; Sigma Aldrich, St. Louis, MO, USA). Cells were culture during 3 days at 37°C in a 48 multiwell plate (Greiner Bio One, Kremsmünster, Austria). Then, supernatants were collected for cytokine quantification.

### Statistical Analysis

Statistical analysis was performed using GraphPad Prism V.8 software (San Diego, CA, USA). The normality of experimental data was evaluated by the Shapiro-Wilk test. Two groups were compared using the Wilcoxon signed-rank test or Mann-Whitney t test as appropriated. Three or more groups were compared using the Kruskall-Wallis test followed by Dunn’s multiple comparison tests. Significance between 2 continuous variables was calculated by using a Spearman correlation test. A p value <0.05 was considered statistically significant.

## Results

### Clinical Characteristics of the Study Population

We recruited a total of 84 girls and boys admitted to different hospitals from Buenos Aires city and surroundings with clinical manifestations of SARS-CoV-2 infection between October 2020 and July 2021. Samples were obtained within 24–72 h of being admitted, after the initiation of therapeutic interventions. Children with COVID-19 included those suffering non-severe (n=67) and severe (n=17) disease. Regarding the non-severe group, the median age (25th–75th percentile) was 3 years (1-7 years), of whom 39% (n=26) were girls. Most children had no co-infections (18%, n=12). Fifty-two percent (n=35) had an existing comorbidity and 6% (n=4) were admitted to pediatric intensive care unit (PICU) due to complications of their underlying disease. Among the severe cohort (n=17), the median age and IQR was 4 years (2-7) and 65% (n=11) were girls. Almost half of them (53%, n=9) had an existing comorbidity. Seventy-six percent (n=13) were admitted to PICU and 47% (n=8) required mechanical ventilation. The median time since onset of symptoms until hospital admission was similar between groups. The characteristics of cohorts are summarized in **Table 1**.

### Children With COVID-19 Showed a Reduced Frequency of Circulating Tregs but an Increased Frequency of TH17 Cells

**Figure 1:**
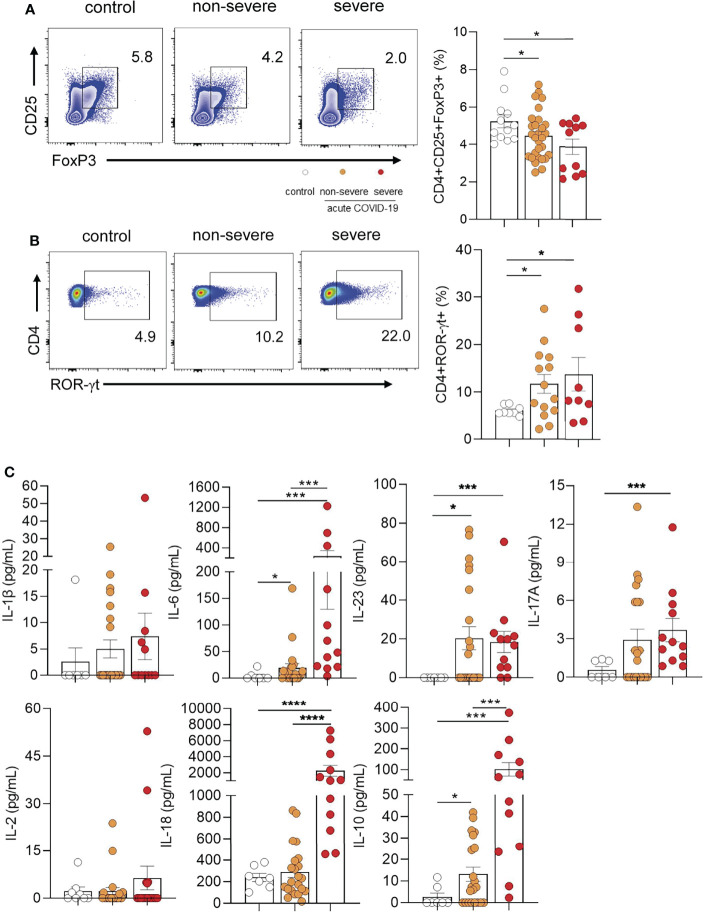
Frequency of Tregs and TH17 cells in children with COVID-19. **(A)** Left panel: Representative dot plot showing the expression of FoxP3 and CD25 in CD4+ T cells. Tregs were defined as CD4+CD25+FoxP3+. Right panel: Frequency of Tregs in controls (n = 12) and children with non-severe (n = 29) and severe (n = 11) COVID-19 analyzed by flow cytometry. **(B)** Left panel: Representative dot plot showing the expression of ROR-γt in CD4+ T cells. Right panel: Frequency of CD4+ROR-γt+ T cells in healthy children (controls, n = 8), and children with COVID-19 (non-severe, n = 14 and severe, n = 9). **(C)** Plasma levels of IL-1β, IL-6, IL-23, IL-17A, IL-2, IL-18 and IL-10 were quantified by multiplex flow cytometric bead array in healthy children (controls, n = 7), and children with COVID-19 (non-severe, n = 22 and severe, n = 12). Data are expressed as the percentage of CD4+ T cells in **(A)** (right) and **(B)** (right). Mean ± SEM of n donors are shown in **(A)** (right), **(B)** (right) and **(C)** P values were determined by Mann-Whitney U test, Kruskal-Wallis test and Wilcoxon test: *p < 0.05, ***p < 0.001, ****p < 0.0001.

By using a combination of anti-CD4, anti-CD25, and anti-FoxP3 antibodies, in a first set of experiments we analyzed the frequency of circulating Tregs by flow cytometry. The gating strategy used is shown in **Supplementary Figure S1**. Representative dot plots are illustrated in **Figure 1A** (left panel). Children with severe and non-severe COVID-19 showed a significant reduction in the frequency of Tregs compared with healthy donors (p<0.05). This reduction was more pronounced in the severe group, however, differences between groups did not reach statistical significance **(** **Figure 1A** right panel).

Because progression of COVID-19 in adults is associated with the induction of a TH17 profile, we evaluated whether this profile was overrepresented in pediatric COVID-19. Representative dot plots of ROR-γt expression (the master regulator of TH17 development) in CD4+ T cells are depicted in **Figure 1B** (left panel). We observed that children with COVID-19 had an increased frequency of CD4+ T cells positive for the expression of ROR-γt compared with healthy donors (p<0.05; **Figure 1B** right panel). Consistent with this finding, cytokine measurement in the plasma from children with COVID-19 showed higher levels of TH17 related cytokines such as IL-6, IL-23, and IL-17A respect to controls (p<0.001). When comparing the levels of these cytokines in severe and non-severe patients, we found no differences for IL-23 and IL-17A. By contrast, plasma levels of IL-6 were markedly augmented in severe patients (p<0.0001). Interestingly, a great increase in plasma levels of IL-18, a cytokine involved in the induction of the CD4+ TH1 profile, was observed in children with severe but not in those with non-severe COVID-19 (p<0.0001). Finally, children with severe disease showed the highest plasma levels of the immunosuppressive cytokine IL-10 (p<0.001). IL-1β and IL-2 were undetectable in almost all donors (**Figure 1C**).

### Extracellular ATP Might be Involved in the Modulation of T Cell Response in Pediatric COVID-19

**Figure 2:**
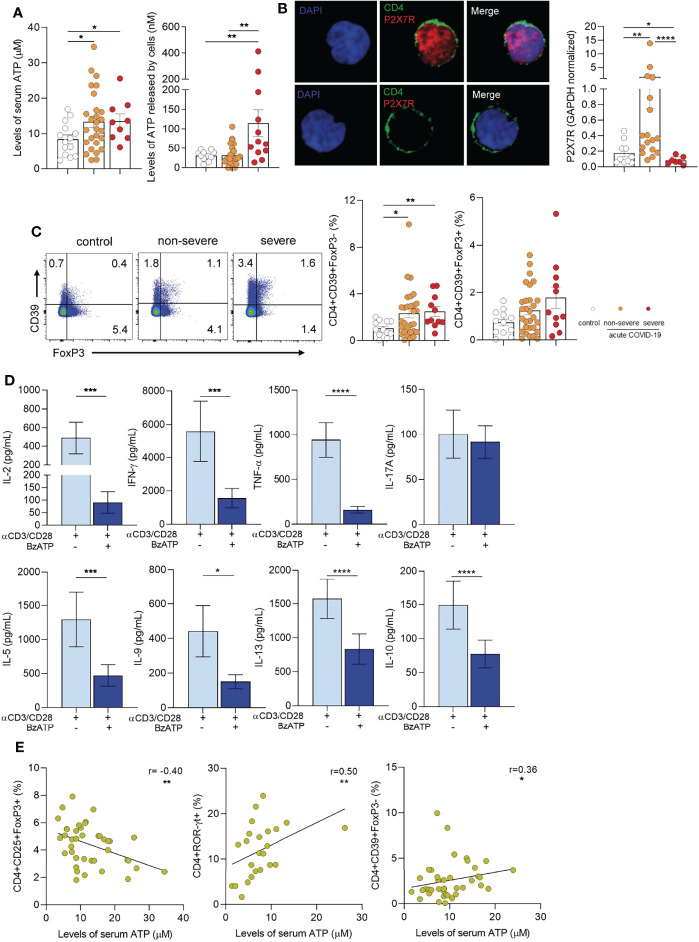
Extracellular ATP-P2X7R axis in children with COVID-19. **(A)** Left panel: Levels of ATP in plasma from healthy children (controls, n = 14), and children with COVID-19 (non-severe, n = 27 and severe, n = 9) measured by luminometry. Right panel: PBMCs **(**5x10^6^/mL) from healthy children (controls, n = 12), and children with COVID-19 (non-severe, n = 25 and severe, n = 12) were stimulated with anti-CD2/CD3/CD28 coated beads (0.3 µg/mL) during 4 min. Levels of extracellular ATP were measured in the supernatant by luminometry. **(B)** Left panel: Confocal microscopy of P2X7R expression in purified CD4+ T cells (green: CD4, red: P2X7R). Nuclear counterstain with DAPI was used. Representative images from a child with non-severe COVID-19 are shown at x300. Right panel: Basal P2X7R expression in PBMCs from healthy children (controls, n = 10) and children with COVID-19 (non-severe, n = 17 and severe, n = 7) quantified by qRT-PCR. **(C)** Left panel: Representative dot plot showing the expression of FoxP3 and CD39 in CD4+ T cells of heathy children and children with COVID-19 (non-severe and severe). Middle and Right panels: Frequency of CD4+CD39+FoxP3- T cells (middle) and CD4+CD39+FoxP3+ T cells (right) in healthy children (n = 12), and children with COVID-19 (non-severe, n = 29 and severe, n = 11). Data are expressed as percentage of CD4+ T cells. **(D)** Purified CD4+ T cells (1×10^6^/mL) from children with COVID-19 (n = 15) were stimulated with anti-CD2/CD3/CD28 coated beads (0.035 µg/mL) and treated or not with BzATP (100 µM) during 3 days. Levels of cytokines were quantified in the culture supernatant by multiplex flow cytometric bead array. **(E)** Graphs showing correlations between the frequency of CD4+CD25+FoxP3+ Tregs (n = 40, left), CD4+ROR-γt+ T cells (n = 24, middle), and CD4+CD39+FoxP3- T cells (n = 40, right) and the levels of ATP in plasma. Mean ± SEM of n donors are shown in **(A, B)** (right), **(C)** (middle and right) and **(D)** P values were determined by Mann-Whitney U test, Kruskal-Wallis test and Wilcoxon test. Correlations were evaluated by using Spearman rank correlation coefficient test. *p<0.05, **p < 0.01, ***p < 0.001, ****p < 0.0001.

Considering previous studies showing that extracellular ATP promotes the development of TH17 cells (Atarashi et al., 2008; Killeen et al., 2013; Pandolfi et al., 2016), we analyzed whether the changes in CD4+ T cell profiles described above were associated to augmented levels of extracellular ATP. We found that plasma ATP levels were higher in children with COVID-19 compared with healthy children (p<0.05). No differences were observed between severe and non-severe patients (**Figure 2A** left panel). To test whether T cells might contribute to the plasma pool of ATP, PBMCs were stimulated with anti-CD2/CD3/CD28-coated beads and the release of ATP was then assessed. We found that PBMCs from children with severe COVID-19 released elevated amounts of ATP compared with non-severe children and controls (p<0.01; **Figure 2A** right panel). Unstimulated PBMCs released almost undetectable quantities of ATP (data non shown).

It has been demonstrated that signaling induced by ATP *via* the purinergic P2X7R promotes T-cell activation (Yip et al., 2009; D’Addio et al., 2018). We confirmed the expression of P2X7R in purified T cells from a child with non-severe COVID-19 by confocal microscopy (**Figure 2B** left panel). We then compared the expression of P2X7R by RT-PCR in PBMCs from heathy children and children with-COVID-19. Interestingly, we found a marked increase in the expression of P2X7R in children with non-severe COVID-19 but not in those with severe-COVID-19 respect to controls (p<0.01, **Figure 2B** right panel), perhaps reflecting a down-regulation in the synthesis of this receptor. We also analyzed the expression of CD39, an ectonucleotidase which mediates the first step in the conversion of ATP into ADP, AMP, and adenosine able to suppress T cell response (Borsellino et al., 2007). Although under physiological conditions CD39 expression is restricted mostly to Tregs, under inflammatory conditions it was also expressed by effector CD4+ T cells (Zhou et al., 2009; Moncrieffe et al., 2010). Representative dot plots of CD39 and FoxP3 expression in CD4+ T cells are illustrated in **Figure 2C** left panel. We observed that FoxP3- but not FoxP3+ CD4+ T cells from children with COVID-19 showed an increased expression of CD39 (p<0.05 and p<0.01 for non-severe and severe vs controls, respectively; **Figure 2C** middle and right panels). Moreover, we found that *in vitro* exposure of purified T cells from children with COVID-19 to BzATP, a P2X7R agonist and ATP analogue, promotes a substantial decrease in a wide pattern of cytokines produced by stimulated T cells without inhibiting the production of IL-17A (**Figure 2D**). Overall, these data suggest that the purinergic signaling might modulate T cell response favoring the induction of a TH17 response. Supporting this possibility, we found that plasma levels of ATP showed an inverse correlation with the frequency of Tregs (r= -0.40, p<0.01; **Figure 2E** left panel). By contrast, we observed a positive correlation between the levels of ATP and the frequency of both CD4+RORγt+ T cells (r=0.50, p<0.01) and CD4+CD39+FoxP3- T cells (r=0.36, p<0.05) as shown in **Figure 2E** middle and right panels, respectively.

## Discussion

Severe COVID-19 in previously healthy children is rare. The reasons explaining the mild and often asymptomatic course of SARS-CoV-2 infection in children are poorly defined. A low expression of host factors involved in the first steps of infection such as the entry receptor ACE-2 and the transmembrane serine protease 2 have been suggested (Bunyavanich et al., 2020; Sajuthi et al., 2020). On the other hand, recent studies propose that children might display a more effective immune response against SARS-CoV-2. It has been reported that children show a pre-activated antiviral innate immunity in the upper airways resulting in a strong expression of genes associated with IFN signaling upon infection (Loske et al., 2021; Pierce et al., 2021; Yoshida et al., 2021). This functional profile appears to control efficiently the early spreading of SARS-CoV-2 infection. Regarding the adaptive immune response, it has been recently published that children develop a robust and sustained cross-reactive spike-specific immune response against SARS-CoV-2, which involves not only a strong antibody response but also a TH1 response. Interestingly, the magnitude of these responses appears to be higher in children compared with adults (Dowell et al., 2022).

The contribution of the different CD4+ T cell profiles to both protective and harmful immune responses to SARS-CoV-2 has been rigorously analyzed in adults with COVID-19 but little is known in pediatric COVID-19. In the present study, we found that COVID-19 in children is associated with a decreased frequency of circulating Tregs and an increased frequency of circulating TH17 cells. Consistent with these findings, we found that plasma from COVID-19 patients displayed an enrichment in TH17-related cytokines such as IL-6, IL-23, and IL-17A. While IL-23 and IL-17A reached similar levels in severe and non-severe patients, IL-6 was markedly heightened in severe patients.

Data regarding Tregs in COVID-19 patients are conflictual. Some studies in adults reported an increased frequency of Tregs displaying an activated phenotype that correlates with disease severity (Neumann et al., 2020; Galvan-Pena et al., 2021; Vick et al., 2021). By contrast, other studies described a reduction in the frequency of Tregs in hospitalized patients (Qin et al., 2020; Wang et al., 2020; Sadeghi et al., 2021). Interestingly, two patients with COVID-19 and acute respiratory distress syndrome were successfully treated with Treg infusion (Gladstone et al., 2020). Only few studies have analyzed this cell subset in children with COVID-19 and the published reports seems to be contradictory; higher frequency (Petrara et al., 2021), lower frequency (Jia et al., 2020), and/or no changes in the frequency of Tregs (Mostafa et al., 2021) have been reported when comparing children with COVID-19 and healthy children. The reasons for the contrasting results are unclear. They could be explained by differences in the characteristics of cohorts as well as in the markers used to define this cell subset. Our results show a moderate but significant reduction in the percentage of circulating Tregs.

The diminished frequency of Tregs that we found in children with COVID-19 is linked to an enhancement in both, the frequency of TH17 cells and the plasma concentration of TH17-related cytokines. These observations are in line with those performed in adults with COVID-19 showing a shift toward a TH17 response (De Biasi et al., 2020; Parackova et al., 2021; Rupp et al., 2021; Sadeghi et al., 2021). In addition, when comparing the plasma levels of cytokines between severe and non-severe children, we found a distinct signature in severe patients characterized by greater levels of IL-6, IL-18 and IL-10. While IL-6 might be a predictive marker for severe disease, IL-18 together with TH17-like cytokines could promote the development of inflammatory responses while inhibiting the differentiation and function of FoxP3+ Tregs. The marked rise in plasma levels of the anti-inflammatory cytokine IL-10 in severe COVID-19 is an unexpected observation, however, a similar finding has been reported in adults with severe COVID-19 (Diao et al., 2020; Islam et al., 2021). It might reflect a physiological response directed to control the development of an over reactive inflammatory response.

It is well-known that ATP accumulates at sites of tissue injury or inflammation. Biological effects mediated by extracellular ATP are mostly mediated through the activation of purinergic receptors (P2 receptors). Among them, the P2X7R expressed by virtually all cells of the innate and adaptive immunity is certainly involved in the induction of inflammatory reactions (Di Virgilio et al., 2017). To our best knowledge, no previous study has analyzed a possible role for the purinergic signaling in pediatric COVID-19. Observations made in adult patients showed higher levels of ATP in the bronchoalveolar lavages from COVID-19 patients compared with healthy individuals (Wauters et al., 2021). Moreover, it has been proposed that P2X7R antagonism could be a promising strategy to prevent or treat neurological complications in COVID-19 patients (Ribeiro et al., 2021). Contrasting with these findings, it was reported that plasma levels of ATP are lower in mild and severe adult COVID-19 patients compared with healthy controls (Dorneles et al., 2021). We here found higher plasma concentrations of ATP in children with severe and non-severe COVID-19 compared with healthy children. Moreover, we found that PBMCs from children with severe COVID-19, but not from children with non-severe disease, were capable of release higher amounts of extracellular ATP upon stimulation with anti-CD3/CD28 coated beads compared with healthy controls, suggesting that activated T cells might contribute to the plasma pool of ATP found in children with COVID-19. On the other hand, our findings revealed a marked increase in the expression of P2X7R in PBMCs from children with non-severe COVID-19. Because the stimulation of P2X7R expressed by CD4+ T cells has shown to promote the differentiation of CD4+ T cells into TH17 and TH1 profiles (Atarashi et al., 2008; Killeen et al., 2013; D’Addio et al., 2018), this might explain, at least in part, the promotion of the TH17 profile observed in children with COVID-19. Interestingly, cells from children with severe disease did not show any enhancement in the expression of P2X7R, but rather they had a lower expression compared with cells from healthy donors. It is intriguing to speculate that this phenomenon might reflect a down-regulation in the production and expression of P2X7R after extensive stimulation by specific ligands, a fact that has already been described (Amadio et al., 2017). Moreover, it might reflect a more general phenomenon related to T cell exhaustion, a phenomenon that has been well defined and characterized in adults with COVID-19 (De Biasi et al., 2020; Heming et al., 2021). Finally, supporting a role for the purinergic signaling pathway in pediatric COVID-19 we found that plasma levels of ATP negatively correlated with the frequency of Tregs and positively with the frequency of TH17 cells.

Our study has a number of limitations. The small number of patients in the cohort of children with severe COVID-19 requires that our observations should be confirmed in large cohorts. A large body of evidence indicate that comorbidity is highly prevalent in severe pediatric COVID-19 (Graff et al., 2021; Ward et al., 2022). Thus, it is not surprising that our hospitalized cohort of children with moderate and severe COVID-19 involves a high percentage of children with underlying disease. These comorbidities might partially explain some of our findings related to the imbalance in the T cell compartment. Another limitation of our study is the unavailability of paired samples from infected children during the acute and convalescent phase. Finally, the small volume of blood collected from each patient impaired to perform studies aimed at characterizing the mechanism through which ATP might modulate T cell function as well as the functionality of Tregs and TH17 cells in children with COVID-19.

In summary, this study offers a novel view of the mechanisms that might control the development of different CD4+ T cell profiles in the course of pediatric COVID-19. Our results show an imbalance in the CD4+ T cell compartment during pediatric COVID-19 and suggest the involvement of extracellular ATP in its induction. Further studies are needed to deeply characterize the mechanisms through which the purinergic signaling modulate the adaptive immune response against SARS-CoV-2 in children with COVID-19 and to define whether extracellular ATP levels could be a biomarker of severity.

## Data Availability Statement

The raw data supporting the conclusions of this article will be made available by the authors, without undue reservation.

## Ethics Statement

Our study was reviewed and approved by the Ethics Committee at the “Hospital de Pediatría Pedro de Elizalde”, Buenos Aires, Argentina, in accordance with the Declaration of Helsinki. Written informed consent was obtained from all donors or legal guardians.

## Author Contributions

Conception and design, CR, JG, and LA. Enrollment of donors, collection of blood sample and clinical data, SR, SA, NC, CD, MS, MB, NS, CB, VN, and FF. Performance of lab experiments, CR, VS, IS, and LA. Analysis and interpretation, CR, VS, IS, JG, and LA. Drafting the manuscript for significant intellectual content, CR, JG, and LA. All authors contributed to the article and approved the submitted version.

## Funding

This work was supported by grants from the National Agency for Promotion of Science and Technology, Argentina (PICTO-COVID-SECUELAS-00007 and PMO BID PICT 2018-2548 to LA).

## Conflict of Interest

The authors declare that the research was conducted in the absence of any commercial or financial relationships that could be construed as a potential conflict of interest.

## Publisher’s Note

All claims expressed in this article are solely those of the authors and do not necessarily represent those of their affiliated organizations, or those of the publisher, the editors and the reviewers. Any product that may be evaluated in this article, or claim that may be made by its manufacturer, is not guaranteed or endorsed by the publisher.

## Abbreviations

ACE-2, angiotensin-converting enzyme 2; Tregs, FoxP3+ regulatory T cells; PBMCs, Peripheral Blood Mononuclear Cells; Bz-ATP, 2´(3´)-O-(4-benzoylbenzoyl) ATP; P2X7R, P2X7 receptor; PICU, pediatric intensive care unit.
