# Supplementary material for: Extracellular ATP and Imbalance of CD4+ T Cell Compartment in Pediatric COVID-19
Source: Front Cell Infect Microbiol. 2022 May 18;12:893044. doi: 10.3389/fcimb.2022.893044 (PMC9157541; doi:10.3389/fcimb.2022.893044)
Supplement: Supplementary file 1 [file DataSheet_1.pdf]

Supplementary Figure 1

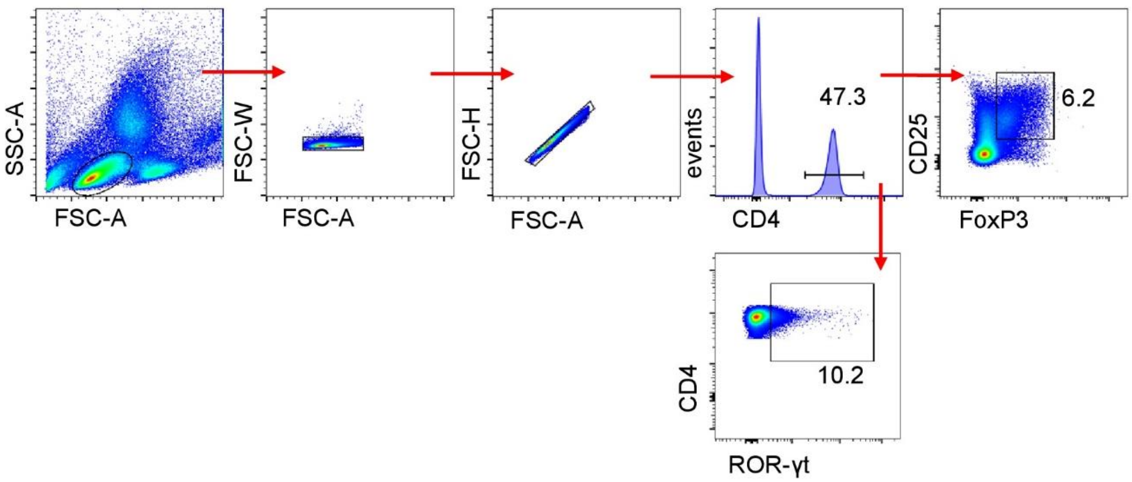

Figure S1. Gating strategy. Representative FACS profile showing the gating strategy for the analysis of Tregs and TH17 cells. Peripheral blood mononuclear cells ($1x10^6$) were stained with anti-CD4 and anti-CD25 IgG antibodies for 20 min at room temperature. Intracellular staining using anti-FOXP3 and anti-ROR-γt was performed on fixed and permeabilized cells. Data were acquired using a FACSCanto II (Becton Dickinson). FSC-A vs SSC-A allowed the preliminary identification of lymphocytes. Then, we selected singlets using FSC-A versus FSC-W and/or FSC-H parameters and CD4+ T cells were selected from the singlet population using the anti-CD4 antibody. Tregs were defined as CD4+CD25+FoxP3+. TH17 cells were defined as CD4+ROR-γt+. A representative experiment is shown.
